# Acute Pancreatitis Secondary to a Perivaterian Duodenal Diverticular Abscess

**DOI:** 10.1155/2010/527141

**Published:** 2010-12-16

**Authors:** P. Pastides, S. Bertaud, S. K. Sarker, S. Dindyal

**Affiliations:** Department of General Surgery, The Whittington Hospital NHS Trust, Magdala Avenue, London N19 5NF, UK

## Abstract

A 46-year-old previously fit lady was admitted with acute pancreatitis. She had no history of gallstones. She was not on any medications and consumed minimal amounts of alcohol. On subsequent investigations as to the causative factor, she was found at ultrasound to have an air-fluid filled cystic structure posterior to the head of pancreas which was compressing the common bile duct. Further magnetic resonance imaging and computer tomography scans showed that this cystic lesion was located around the ampulla of Vater. A diagnosis of a perivaterian abscess was made. At endoscopy, a large contained abscess was seen which was successfully drained. She made a full and uneventful recovery.

## 1. Introduction

We present a case of acute pancreatitis caused by a duodenal diverticular abscess occluding her ampulla of Vater. This case report is the first documented case in the literature of acute pancreatitis caused by a perivaterian duodenal diverticular abscess.

## 2. Case Presentation

A thirty-eight-year-old female presented to our emergency department with one-day history of acute onset epigastric pain. It was constant and sharp in nature and associated with several episodes of vomiting. There was no history of fevers, rigors, dysuria, or change in bowel habit. She gave a two-week history of preceding mild abdominal pain, particularly after eating. She was previously fit and well with no history of gallstones, took no regular medications, and had no significant past medical history. She had no known drug allergies and was on no regular medication. She was a nonsmoker and consumed around four units of alcohol per week.

On inspection, she looked unwell. She was apyrexial and tachycardic. On palpation of her abdomen, she had marked epigastric tenderness and guarding. Her blood tests revealed a raised white cell count (16.5), bilirubin (26), ALT (165), AST (222), LDH (494) and amylase level of 2245. She was diagnosed with acute pancreatitis, scoring 2 on the Glasgow severity score.

**Figure 1:**
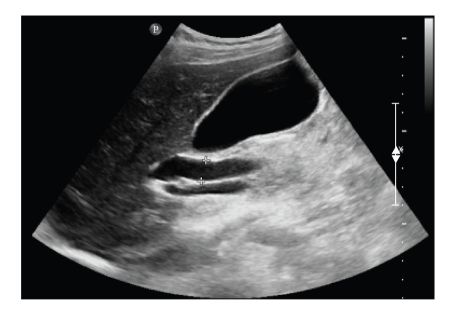
Abdominal ultrasound showing a dilated common bile duct (11 mm).

An abdominal ultrasound demonstrated a dilated common bile duct (11 mm) with a distended gallbladder with no gallstones or other pathology (Figure 1). 

**Figure 2:**
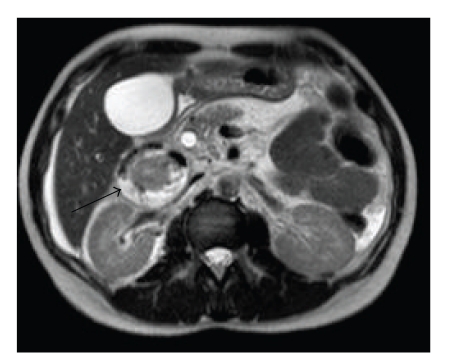
MRCP slice showing an air-fluid filled cyst (arrowed) posterior to the head of the pancreas.

A MRCP performed the following day confirmed a 12.4 mm dilated bile duct with dilation of the intrahepatic biliary tract. In addition, it revealed a “curious” cystic lesion, with an air-fluid level, lying posterior to the head of the pancreas at the level of the distal common bile and pancreatic duct, which appeared to be causing some extrinsic compression (Figure 2). 

**Figure 3:**
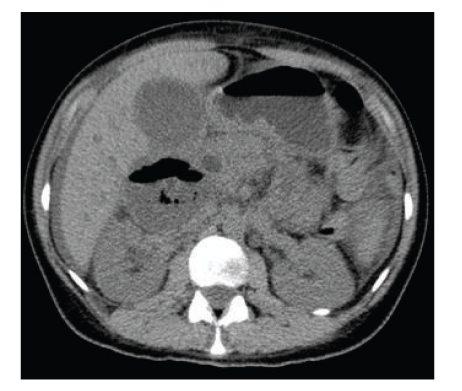
CT scan slice showing the air-fluid filled cyst.

She began to improve clinically and haematologically (reduced leukocytosis) with oral antibiotics. However her bilirubin continued to rise, peaking at 58. She underwent a CT scan of her abdomen on her fourth day of admission. This showed a cystic or necrotic mass behind the head of the pancreas, representing an inflammatory lesion, phlegmon, or duodenal diverticular abscess (Figure 3).

**Figure 4:**
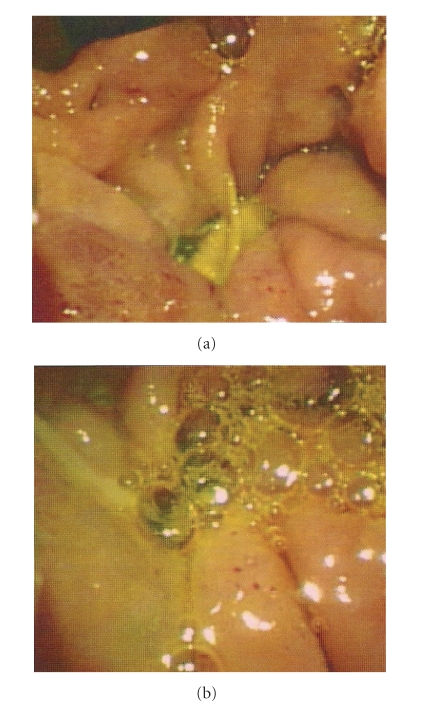
Images from the ERCP showing a duodenal ulcer which was draining pus and bile.

An ERCP on her sixth day postadmission revealed a 10 cm duodenal diverticular abscess draining pus and bile, at the level of the major papilla (Figure 4). It appeared that the cause of her pancreatitis was extrinsic compression of the bile duct due to a perivaterian duodenal diverticular abscess. 

## 3. Discussion

This is the first documented report of a perivaterian diverticular abscess causing acute pancreatitis due to compression of the ampulla of Vater. 

Duodenal diverticula occurs with a frequency of between 5% and 25% [1]. The large variance in these figures is due to the fact that they tend to be asymptomatic and mostly diagnosed if they cause complications or at autopsy. They are mostly located around the posterior border in the second part of the duodenum. Those located around the ampulla of Vater, as described in this patient, are known as perivaterian diverticular abscesses [2]. 

Recognised complications of these types of diverticulae include mechanical obstruction and perforation leading to peritonitis, requiring urgent surgical intervention. Ulceration giving rise to upper gastrointestinal bleeding has also been reported and could be fatal if the erosion involves the aorta or a mesenteric vessel [3, 4].

Radiological diagnosis of these abscesses can be difficult. Since the commonest site of formation is at the second part of the duodenum, a large fluid-filled cystic lesion could easily be diagnosed as a neoplasm of the pancreas arising from the head of the pancreas, which is the common site of pancreatic neoplasm formation. Computer tomography and magnetic resonance imaging scans are useful to distinguish between these two widely varying diagnoses by demonstrating characteristic air-fluid levels within these lesions [5, 6]. Upper gastrointestinal endoscopy has been shown in various studies to be a useful diagnostic tool; however, if the diverticula are located in the third or fourth part of the duodenum, then the sensitivity decreases [7].

It has been shown that there is an association between periampullary diverticula, which can lead to abscess formation, and biliary duct stones. However a large study showed that there is no association between periampullary diverticula and pancreatitis [8]. Thus in a patient suffering from pancreatitis with dilated bile ducts but no gallstones, the diagnosis of perivaterian abscess should be considered. 

Our presented patient was successfully treated with endoscopic drainage of this abscess and made a full uncomplicated recovery. She was seen four months postoperatively in clinic with no reported problems. She is now being routinely followed up at our local tertiary hepatobiliary centre. Endoscopic relief for similar cases has been reported successfully in several other cases [8, 9] and is an alternative to more invasive procedures, with the advantage of faster recovery times for patients. 

## 4. Conclusion

This is the first documented case of a perivaterian duodenal abscess causing compression of the common bile and pancreatic duct leading to pancreatitis. Duodenal diverticula are more common than previously thought, but case reports are scarce as the majority are silent and cause no significant clinical manifestations. Care must be taken to diagnose the condition correctly by using appropriate imaging modalities. Successful endoscopic treatment is possible and should be attempted in appropriate patients.
